# Music Appreciation of Cochlear Implant Users versus Normal Hearing Individuals

**DOI:** 10.22038/IJORL.2022.62651.3152

**Published:** 2022-05

**Authors:** Azam Nasresfahani, Shayan Dasdar, Nika Kianfar, Masoud Motasaddi Zarandy, Farzad Mobedshahi, Sasan Dabiri, Ali Kouhi

**Affiliations:** 1 *Department of Cochlear Implant Center and Otorhinolaryngology, Amir-A'lam Hospital, Tehran University of Medical Sciences, Tehran, Iran.*

**Keywords:** Appreciation, Cochlear Implant, Listening habits, Music, Postlingual, Prelingual

## Abstract

**Introduction:**

Cochlear implants (CI) provide speech perception for patients with sensorineural hearing impairment; nonetheless, listening to music is a daunting challenge for them. The present study aimed to compare Iranian CI users and normal hearing (NH) controls in terms of musical habits and appreciation and investigate the possible effect of background variables.

**Materials and Methods:**

A total of 37 CI users who underwent surgery at least 18 months before the study and 59 NH listeners were enrolled in this study. The participants were assigned to two age groups: group A (patients ≥15 years old) and group B (patients <15 years old). They were asked to complete the questionnaires to assess their music engagement.

**Results:**

In group A, the mean score of music importance was significantly higher in CI users (8.7±2.1), as compared to that in NH subjects (5.8±2.3) (P=0.005). Participation in professional musical training and singing with music was not significantly different between the groups. In group B, the mean score of desire for music was not significantly different between CI users (8.2±1.8) and NH subjects (7.7±2.0). They participated in professional musical training and had a reaction to music almost equally. Singing with music was significantly less common in the CI group (CI 16[61.5%], NH (40[85.1%]) (P=0.023). Selected background variables had no significant effect on the music tendency and habits of CI users.

**Conclusions:**

Iranian CI users tended to have a high level of music appreciation in both adult and children groups. Moreover, CI users and NH controls did not significantly differ in the importance of music, devoted time, participation in musical activities, and musical habits.

## Introduction

Music is an inseparable part of human culture, improving physical health, apart from its substantial benefits for mental well-being (1). Immune system promotion (2), better recovery after stroke (3), and pain-reducing effect in patients with cancer (4) are some physiological benefits of music. Nonetheless, some people are deprived of these advantages due to hearing disabilities. With the introduction of the cochlear implant (CI) and its subsequent developments, this technology became the standard treatment for patients with profound sensorineural hearing loss (5). Cochlear implantation improved the quality of life of patients in different aspects, especially communication, social activities, and not feeling like a burden (6-9). Although the improvement in speech perception was so remarkable, more difficult hearing tasks, such as music perception, have remained challenging (10-12). The CI users have a poorer musical admiration, compared to normal hearing (NH) individuals, and they generally perceive music as out-of-tune, discordant, and emotionless (13). Better music perception was observed among patients who were more involved in musical experiences (14-16). Nevertheless, participation in music activities depends on whether music is still important for them. Several studies have evaluated the music appreciation of CI users, yielding various findings (17-23). Regarding music engagement of CI users, the majority of previous studies were performed based on Western music.

In Iranian culture, music has been integrated with people’s everyday activities for centuries. In comparison with Western music, Iranian traditional music is more vocal and unique for having a combination of quarter tones. Different cultures and habits of Iranian people may affect the musical experiences of CI users; nonetheless, no study has evaluated this issue yet. In light of the aforementioned issues, the present study aimed to assess musical habits and tendencies in Iranian CI users, as compared to those in NH controls.

## Materials and Methods

***Study Design***

This cross-sectional case-control study was conducted at Amir Alam Hospital, a tertiary referral center for cochlear implantation in Iran. The study protocol was approved by the Research Institutional Review Board of Tehran University of Medical Sciences.

***Participants***

The subjects of this study were a convenience sample of patients with cochlear implantation who were referred for regular follow-up to our clinic. The inclusion criteria entailed speaking in Persian, a minimum of 18 months of experience with CI, and willingness to participate. Patients were evaluated in two separated age groups: ≥15 years old (group A) and <15 years old (group B). It is noteworthy that 15 years of age was selected as a cut-off since individuals after this age (the age of high school in Iran) become more independent from the family and their personal musical habits are formed. Moreover, NH controls were selected among patients’ relatives to diminish the potential confounding effects of culture and socioeconomic level. 

The inclusion criteria for NH listeners were as follows: first- or second-degree relatives of CI users, a maximum age difference of 3 years old, and no history of hearing loss. This method of control identification can reduce the differences between groups and increase the response rate and validity of the data. Moreover, as illustrated in previous studies, the musical experiences of individuals are heavily tied to their families; therefore, this design would eliminate the possible impact of family differences (20,24). All volunteer individuals who completely met the above condition were recruited as NH controls.

***Questionnaire***

The primary purpose of this study was to compare CI users and NH listeners in terms of the importance of music, participation in musical activities, and music listening habits. Most instruments of subjective measure for music engagement were in English, and there was no validated Persian questionnaire available in this field. Among the feasible questionnaires, we found Munich Music Questionnaire (MUMU) more relevant to our purpose, although MUMU was created only for the post-lingually adult CI users (25). Therefore, we needed to create a new culturally adapted Persian instrument, which could cover all the objectives of the current study. The majority of this questionnaire was adopted from MUMU. Two different questionnaires were designed for groups A and B. Three experienced audiologists reviewed the first version of each questionnaire and based on their feedback regarding the clarity and length of the questionnaires, the latest version was prepared. Each questionnaire consisted of two parts. The second part was filled out only by CI users exploring hearing loss, cochlear implantation, use of hearing aids, resumption of musical activity, and device information. A blank copy of the questionnaires is provided in Appendix A. For group A, the questionnaire investigated the following features of musical engagement: the amount of time devoted to music (item 1), the overall importance of music (none=0 to very much=10) (item 2), music listening habits (items 3-6), music listening preferences (items 7-11), professional musical training (formal training for more than six months) (item 12), singing (item13), and family involvement in music (item 14). For group B, a more concise questionnaire was developed to assess the child’s desire for music (none=0 to very much=10) (item 1), reaction to music (item 2), music listening preferences (items 3-6), professional musical training (formal training for more than six months) (item 7), singing (item 8), and family involvement in music (items 9-11). In group B, questionnaires were filled out by patients’ parents; nonetheless, they were asked to do it in the presence of children. 

***Evaluation of music appreciation***

For group A, music appreciation was evaluated 

by items 1,2,12, and 13, while in group B, music appreciation was assessed by items 1,2,7, and 8. Moreover, we assessed the possible effect of background factors on music appreciation in rehabilitated deafened patients. Background variables included the following items: age at implantation, deafness span, duration from implantation to study, CI’s model, number of electrodes, utilizing hearing aids before implantation, and post-implantation hearing level.

***Statistical analysis***

Statistical analysis of the data was performed using the Chi-square test to compare categorical variables and an independent-sample t-test for continuous variables between NH and CI groups. Moreover, linear regression and independent-sample t-test were used to assess the effect of background factors on the music appreciation of CI users. The data were analyzed in (version 24.0). A p-value of less than 0.05 was considered statistically significant.

## Results

***Group A***

*Matching analysis*

This section of the study included two groups of patients (n=11) and NH controls (n=12). The matching features between CI and NH groups were based on personal characteristics and family-social status (Table1). Statistical tests indicated that there was no significant difference between the two groups regarding age, gender, education, and professional musical training in the family, signifying their uniformity of them in the assessment of music appreciation.

**Table:** Matching Analysis between normal hearing (NH) subjects and cochlear implant (CI) users in patients ≥15 years old (group A)

**Matching Factors**		**CI**	**NH**	**P value**
Age(years)		20.27±1.42	22.25±3.62	0.101
Gender, n (%)	Male	8 (72.7%)	5 (41.6%)	0.214
	Female	3 (27.3%)	7 (58.3%)	
Education level, n (%)	Bachelor’s degree or lower	5 (45.5%)	8 (75%)	0.414
	Master’s degree or higher	6 (54.5%)	4 (25%)	
Professional musical training in family*, n (%)	yes	6 (54.5%)	7 (58.3%)	1.000
	no	5 (45.5%)	5 (41.6%)	

* Formal training of at least one of the first-degree relatives for more than six months

***Demographic features***

Out of 11 patients, 9 (81.8%) and 2 (18.2%) cases were pre-lingually and post-lingually deafened, respectively. All the included patients had a unilateral CI, 4 (36.4%) right-sided and 7 (63.7%) left-sided. Moreover, 6 (54.6%) cases were implanted before 3 years of age, and the other 5 (45.5%) subjects were implanted at older ages. In 5 (45.5%) patients, the musical experience resumed instantly after their implantation, and in 6 (54.6%) subjects, it took more than 2 years. The mean duration from implantation to study was 14.7 years (range: 4-20 years). Moreover, 8 (72.8%) patients used Clarion (Advanced Bionics, Sylmar, CA, U.S.A) with 10 electrodes and only 1 (9.1%) case used Nucleus Contour (Cochlear Americas, Englewood, CO, U.S.A) consisting of 24 electrodes. The self-reported hearing level was excellent in 6 (54.6%) patients and good in 4 (36.4%) subjects. Out of 11 patients, 6 (54.6%) cases had used hearing aids before their implantation. 

***Music appreciation***

Regarding the collected data, most respondents in the CI group selected “more than 5 hours” in a week (CI 4[40%], NH 1[8.3%]) (Figure 1). The mean score of music importance was significantly greater in CI users (mean ± SD: 8.72±2.05), compared to that in H subjects (mean±SD: 5.83±2.33) (P=0.005). The number of cases with professional musical training was not considerably different between the two groups (CI 4[36.4%], NH 6[50%]) (P=0.680). Singing with music was not significantly different between CI users 6(54.5%) and NH subjects 5(41.7%) (P=0.511).

**Figure:**
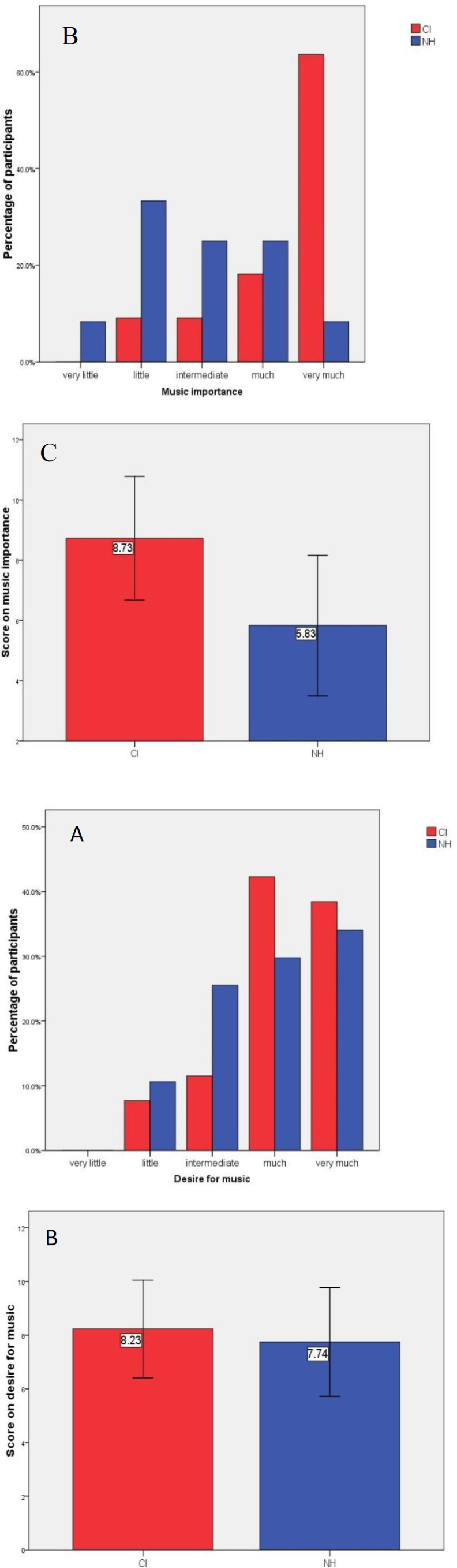
**Answers of participants aged ≥ 15 years old (group A) to A) how much they listen to music, B) how much music is important, and C) the mean score of music importance** **(p=0.005)** **in the two groups of cochlear implant users and normal hearing (NH) subjects**

*Musical habits*

The distribution of musical habits was the same between CI users and NH subjects. In both groups, the preferred place for listening to music was home (CI 3[27.3%], NH 6[50%]), the preferred device was a cell phone (CI 6[54.5%], NH 8[66.7%]), the reason of listening was relaxation (CI 6[63.6%], NH 10[83.3%]), and the most pleasant music genre was pop (CI 8 [72.8%], NH 10[58.3%]), followed by Iranian traditional music (CI 7[63.6%], NH 6[50%]). All CI users listened to music through the CI microphone.

The CI users preferred to listen to music without any other avocations (CI 5[45.5%], NH 1[8.3%]) and low-pitched melodies (CI 4[36.4%], NH 2[16.7%]) in a way that none of them liked high-pitched music. Based on the data, the number of instruments and the presence of singers in melodies made no difference in musical habits in both groups.

***Background variables impact***

No significant association was observed between music appreciation and each selected background variable. 

The musical tendency was assessed by the following items: quantity of listening to music in a week, the score of music importance, having professional musical training, and singing along with music.

 ***Group B***

*Matching analysis*

A total of 73 participants, 26 CI users and 47 NH subjects, were included in this part of the study. The subjects in the two groups were homogeneous in personal characteristics and family-social status. There was no statistically significant difference between the two groups regarding age, gender, education level, history of maternal listening to music, and lullaby listening. Nonetheless, musical training in the family was significantly more common in NH participants (Table 2).

**Table:** Matching Analysis between normal hearing (NH) subjects and cochlear implant (CI) users in patients <15 years old (group B)

**Matching factors**		**CI**	**NH**	**P value**
Age(years)		7.58±3.276	8.38±3.083	0.299
Gender, n (%)	Male	10 (38.5%)	24 (51.1%)	0.301
	Female	16 (61.5%)	23 (48.9%)	
Education level†, n (%)	Bachelor’s degree or lower	20 (76.9%)	32 (69.6%)	0.503
	Master’s degree or higher	6 (23.1%)	14 (30.4%)	
Professional musical training in family*, n (%)	yes	3 (11.5%)	19 (41.3%)	0.008
	no	23 (88.5%)	27 (58.7%)	
Maternal listening to music, n (%)	yes	11 (42.3%)	15 (31.9%)	0.375
	no	15 (57.7%)	32 (68.1%)	
Lullaby, n (%)	Yes	19 (73.1%)	37 (78.7%)	0.585
	No	7 (26.9%)	10 (21.3%)	

† The most education level among parents was considered.

*Formal training of at least one of the first-degree relatives for more than six months

***Demographic features***

Out of 26 patients, 18 (61.2%) and 8 (30.8%) cases were pre-lingually and post-lingually deafened, respectively. Among these patients, 25 (96.1%) cases had a unilateral CI (22[84.6%] right-sided and 3[11.5%] left-sided), and 1 (3.9%) patient had bilateral CIs. Moreover, 12 (48%) subjects were implanted before the age of 3, and 13 (52%) cases were implanted at older ages. The deafness duration before implantation ranged from 2-10 years (mean±SD: 3.61±1.96), and the duration of CI usage ranged from 2-8 years (mean±SD: 4.00±1.94). 

Nucleolus Freedom (Cochlear Americas, Englewood, CO, U.S.A) which consisted of 24 electrodes was the most used device (18[75%]), followed by Nucleus Contour (Cochlear Americas, Englewood, CO, U.S.A) (5[20.8%]) with 24 electrodes. Furthermore, 24 patients had used hearing aids before implantation (92.3%). The self-reported hearing level was good in 14 (53.8%) cases and excellent in 12 (46.2%) patients.

***Music appreciation***

The mean score of desire for music in NH (mean ± SD: 7.74 ± 2.03) and CI (mean ± SD: 8.23 ± 1.82) groups was not significantly different (P=0.457) (Figure 2). Subjects in both groups had reaction to music equally (CI 24[92.3%], NH 47[100%]) (p=0.124). Moreover, 21(80.8%) CI users and 40 (85.1%) NH subjects had professional musical training (P=0.744). Singing with music was significantly less common in the CI group (16[61.5%]), compared to that in the NH group (40[85.1%]) (P=0.023).

**Figure:**
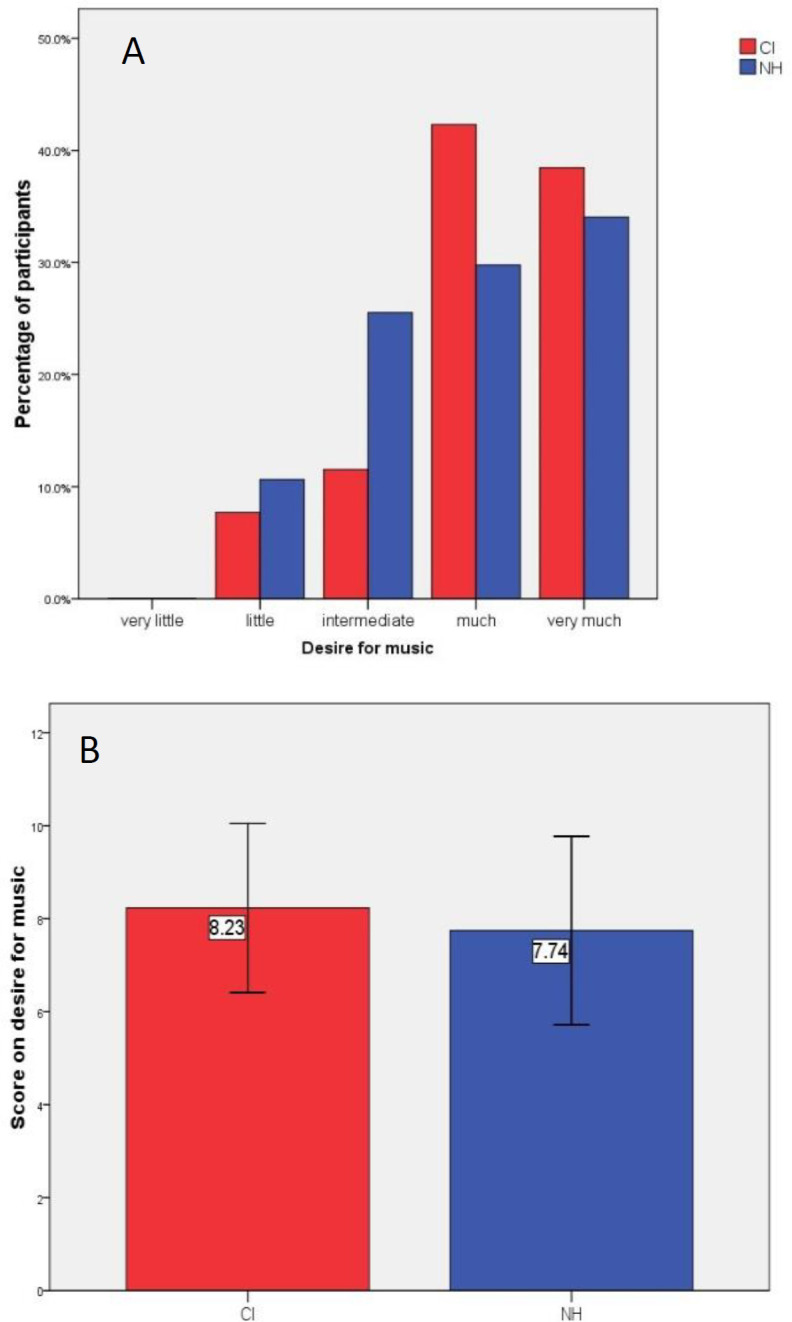
Answers of participants aged <15 years old (group B) to A) how much desire the child has for music and B) the mean score on a desire for music (P=0.457) in the two groups of cochlear implant users and normal hearing subjects

***Musical habits***

Regarding musical habits in both groups, most of the subjects preferred listening to music in uncrowded locations (CI 11[42.3%], NH 17[36.2%]), were interested in happy music (CI 19[73.1%], NH 36[76.6%]), and preferred music with a singer (CI 10[38.5%], NH 25[53.2%]). In all CI patients, the CI microphone was used for listening to music. 

***Background variables impact***

No significant association was observed between music appreciation and background variables. Music appreciation was assessed by the following items: score of desire for music, having a reaction to music, having professional musical training, and singing along with music.

## Discussion

Music is one of the essential components of human culture, promoting different aspects of life. Listening to music can provide both physiological and psychological benefits (1). It has been demonstrated that patients who had undergone cochlear implantation had difficulties in music perception. Nonetheless, another important point to investigate is how CI users’ tendency for music changes after implantation, particularly, if listening to music continues to be important for them. This study made a comparison between CI users and age, gender, and family-social status matched NH controls in terms of music appreciation.

There is a growing body of literature regarding how the music involvement of CI recipients alters after implantation. in a review study, Looi et al. (18) stated that CI users dedicated less time to musical experiences after implantation, compared to before deafness, and some of them fully avoided music. On the contrary, Migirov et al. (17) reported that despite the noticeable post-implantation reduction in music listening time, most CI users resumed their musical experiences. 

In a similar vein, Drennan et al. (21) observed a decrement in desire for music and stated that more music listening hours were associated with higher perceptual abilities. The retrospective design of these studies might have led to an exaggerated judgment of respondents regarding the reduction in music listening time. 

Therefore, further studies were conducted to compare music experiences between CI users and NH controls. In a study by Adams et al. (19), CI recipients obtained lower scores in the frequency of music listening and enjoyment; however, the two groups were not matched for age, and NH participants were significantly younger. Bruns et al. (22) found that music appreciation was not different between pre-lingually deafened CI users and NH subjects although it was significantly lower in post-lingually deafened CI users. Dritsakis et al. (23) also reported an equal level of music importance in CI users and NH peers; nonetheless, CI users were less frequently engaged in musical activities.

The results of the present study indicated that CI users in group A devoted comparable amounts of time to NH subjects, and interestingly, music seemed to be more important to them. A comprehensive view of various features of music engagement (including devoted time, importance score, singing, and participation in musical activities) points to a satisfactory level of music appreciation in adult CI users in this study. This finding can be ascribed to the fact that the majority of our patients were pre-lingually deafened (9 out of 11), and as illustrated, the lack of prior musical experience can increase music acceptance in pre-lingually deafened patients (17, 26). The special features of Iranian music and culture might be another reason that contributed to the higher appreciation of music among CI users.

Iranian traditional music is based on a modal system with a crucial role of vocalists. The frequency range in most Iranian music is almost equal to the vocal tone range. Moreover, most Iranian instruments cover the range of no more than three octaves by taking advantage of microtones. In Iranian culture, music, especially in the form of vocal music, has a substantial presence in childhood. Mothers’ lullabies, poetry teaching, and participation in vocal ensembles are some common examples of child engagement in music. Therefore, Iranian children can benefit from these exclusive features during their growth and development. In group A, no obvious difference was detected in musical habits between CI and NH subjects. The data have indicated that the desired location, music type, commonest device, and the reason for music listening were same between CI users and NH subjects. However, in agreement with the previous studies, CI users preferred focused listening and low-pitched melodies (10,13). In the second part of the study, we evaluated the music appreciation of children. The analysis confirmed that CI users in group B were fascinated by music as much as NH subjects, and they had the same rate of reactions to music. Both groups participated in professional musical activities equally. Nonetheless, singing was a very rare phenomenon among CI users in group B which might be due to their disability to hear their own voices clearly. The provided data regarding children might vary in precision due to personal characteristics, emotions, and expectations of the parents. Nevertheless, the similarity of music involvement between children CI users and NH siblings was reported in the study by Driscoll et al. (20) as well. Altogether, it can be stated that a child’s interest in music is preserved after implantation. Some of the identified factors that can enhance the music engagement of children are family involvement in music and better pitch perception (20,24).

In group B, musical habits, including preferred location, music type, and the presence of singers, were the same between CI users and NH participants. In the present study, background variables had no significant effect on music appreciation in groups A and B. Recently, Gfeller et al. (27) evaluated the personal characteristics and experiences of CI users who were successfully engaged in musical activities. They noticed that the capacity to persist with music exercises despite the annoying sound was the most prominent factor contributing to their success. As illustrated in the current study, typical CI users in both age groups also had a remarkable music appreciation. This finding implies that if hearing professionals provide a suitable rehabilitation program that enhances the skills and motivations of CI users, there would be a sufficient willingness among the patients to participate. The first limitation of this study was the small sample of adult patients. Although cochlear implantation has a long history in Iran, the number of patients was limited in the first decade when implantation was introduced. Another limitation was the wide range of deafness duration in children CI users which might affect the residual hearing and music tendency of participants. This wide range might be due to the challenges, such as high cost and a lack of CI centers, posed to CI candidates in Iran (28). Moreover, although small changes were made to the main MuMU questionnaire, its reliability and validity should be assessed. Despite the aforementioned factors, sufficient data were yet provided to demonstrate the appreciation of music in CI users. Further studies with larger sample size should be conducted to evaluate Iranian music features, along with the perceptual abilities of CI users.

## Conclusion

As evidenced by the results of this study, music importance, devoted time, participation in musical activities, and musical habits were not different between CI users and NH controls, and even in some aspects, CI users reported a higher score. These findings can demonstrate that both adults and children Iranian CI users appreciated music at least as much as NH listeners do. Indeed, music appreciation of CI users can ensure their participation in rehabilitation programs.
